# Supplementary figures and images for: Screening of transporters to improve xylodextrin utilization in the yeast Saccharomyces cerevisiae
Source: PLoS One. 2017 Sep 8;12(9):e0184730. doi: 10.1371/journal.pone.0184730 (PMC5591001; doi:10.1371/journal.pone.0184730)

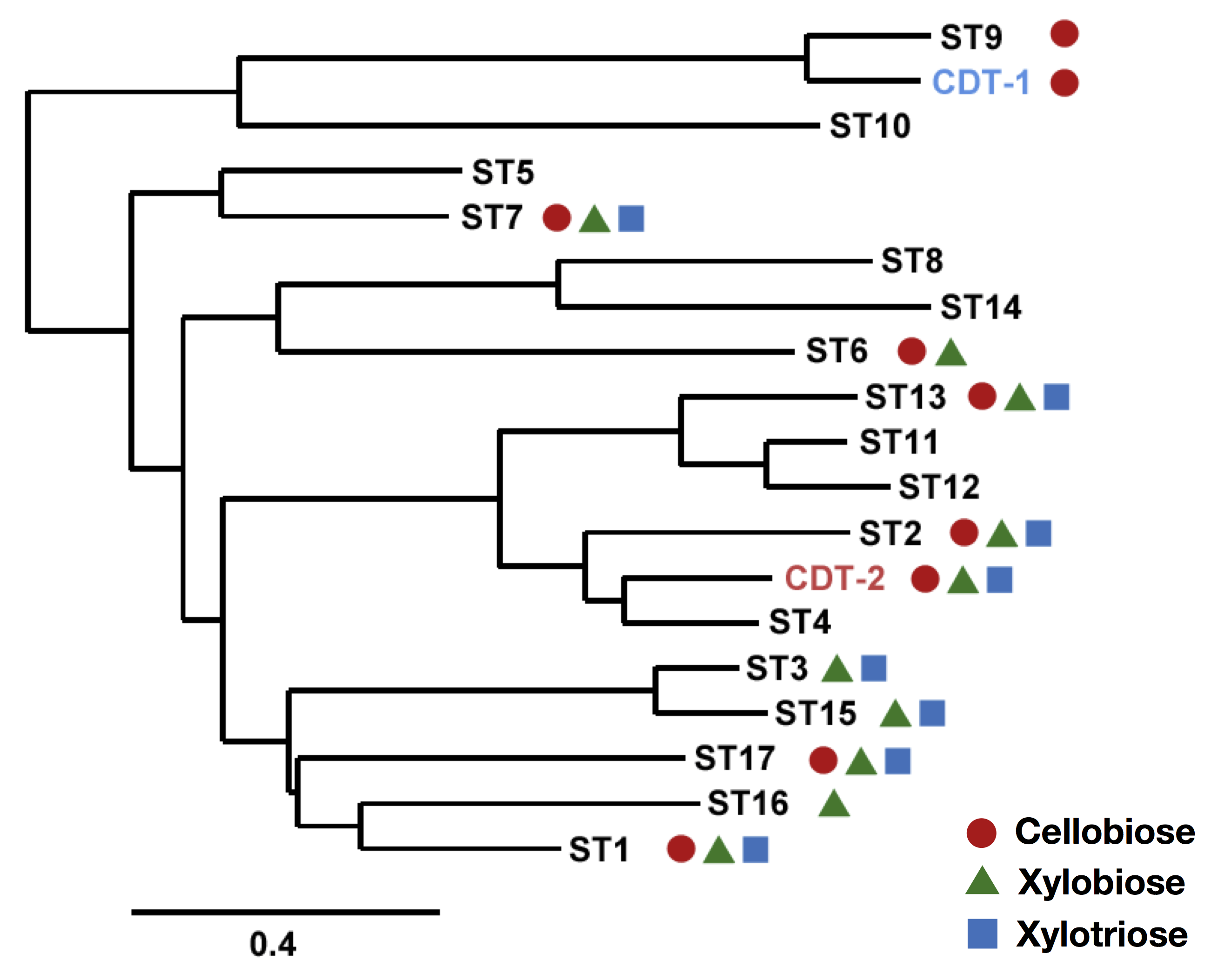

Supplement: S1 Fig — Phylogenetic tree of ST transporters showing the substrates for each transporter: cellobiose, xylobiose, and xylotriose. (TIFF) [file pone.0184730.s001.tiff]

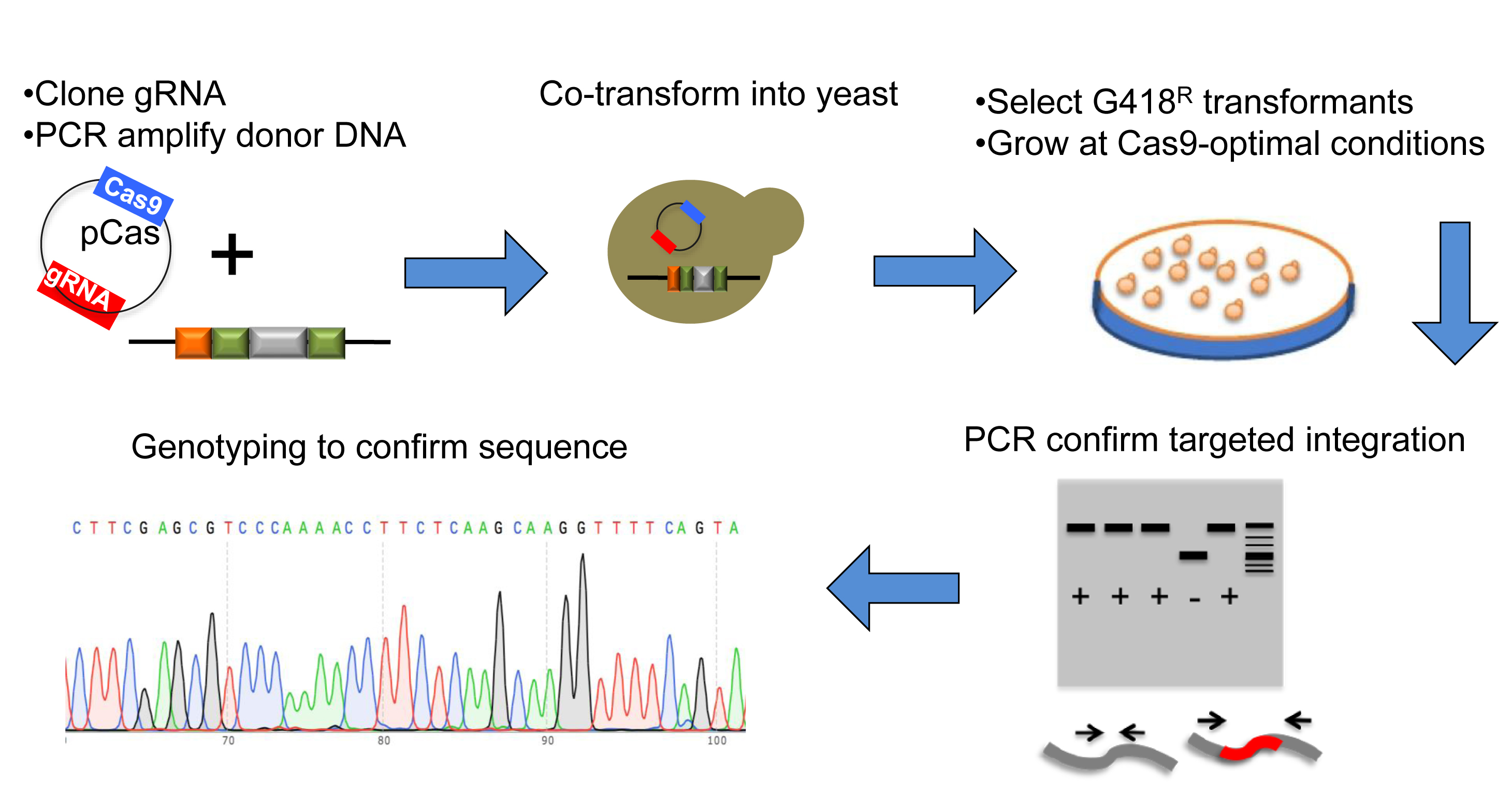

Supplement: S2 Fig — pCas plasmid with Cas9 and sgRNA and double strand repair DNA are co-transformed into yeast. Cells harboring pCas were G418 resistant. PCR of the G418R colonies was used to confirm targeted integration, and genotyping was used to confirm sequence. (TIFF) [file pone.0184730.s002.tiff]

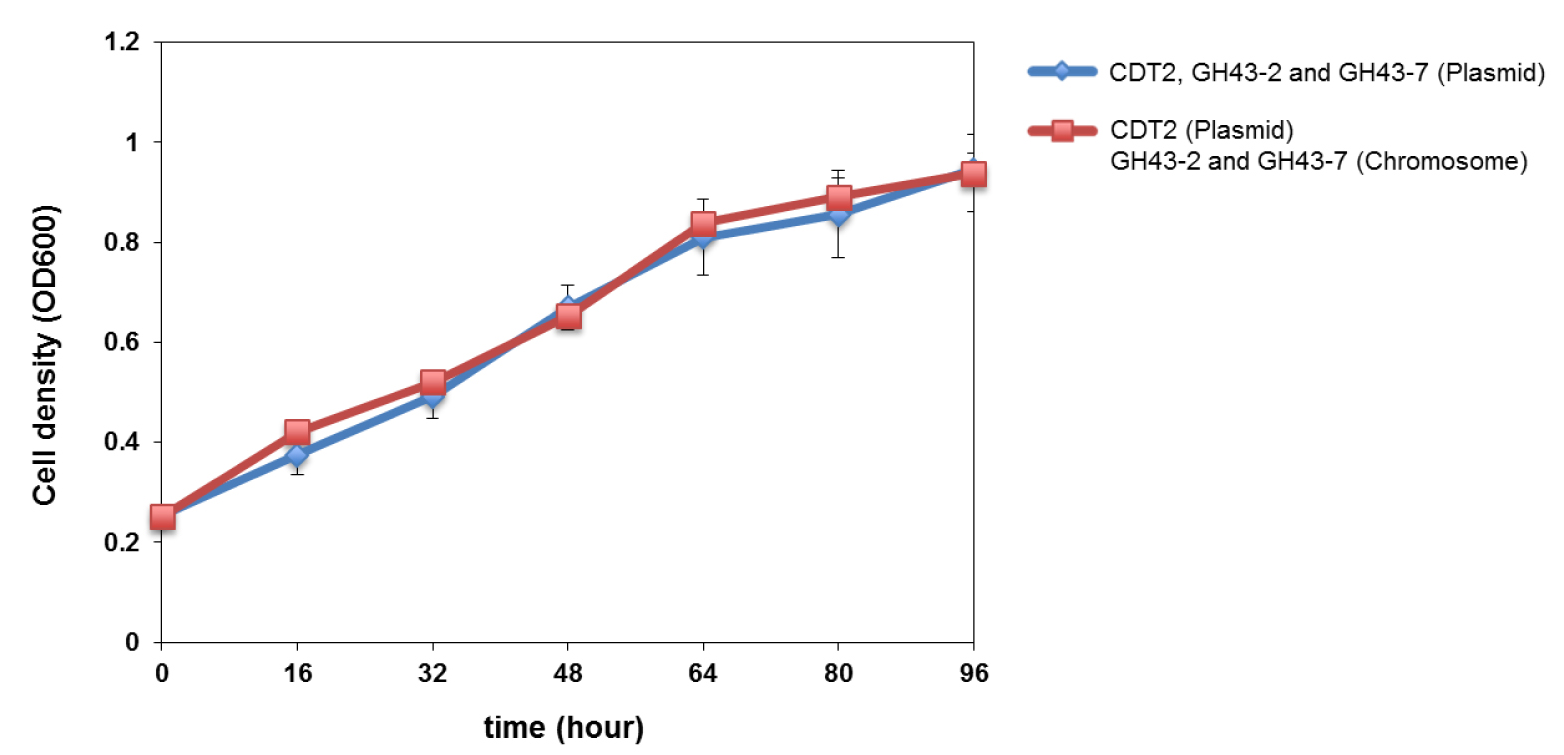

Supplement: S3 Fig — Yeast strain SR8 with a plasmid expressing three genes (CDT-2, GH43-2 and GH43-7) and SR8A with GH43-2 and GH43-7 expressed from chromosomally-integrated genes, and a plasmid only expressing the CDT-2 transporter are shown. Error bars represent standard deviations of biological triplicates. (TIFF) [file pone.0184730.s003.tiff]

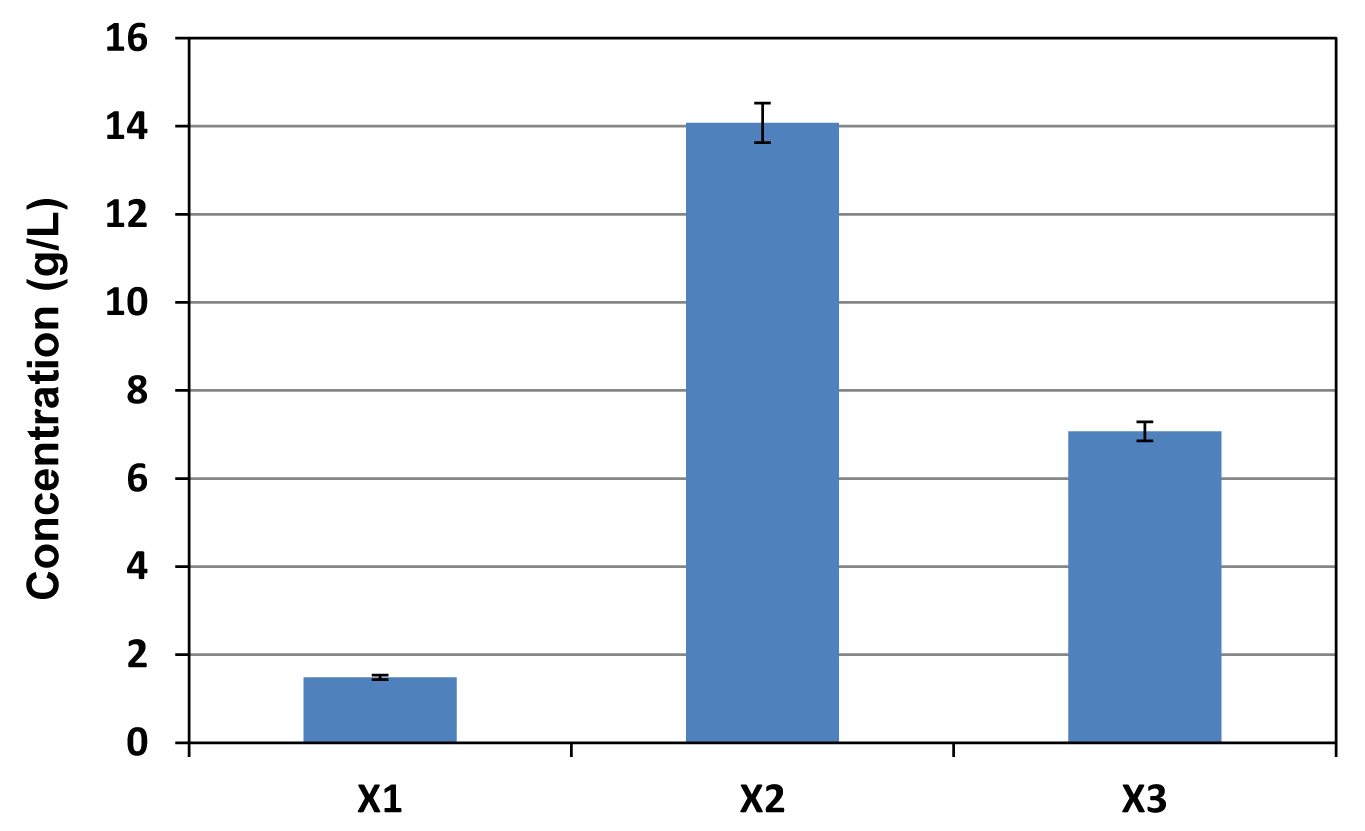

Supplement: S4 Fig — Carbohydrate levels seen in chromatograms in the yeast growth media are shown with error bars representing standard deviations of biological triplicates. Compounds are abbreviated as follows: X1, xylose; X2, xylobiose; X3, xylotriose. (TIFF) [file pone.0184730.s004.tiff]

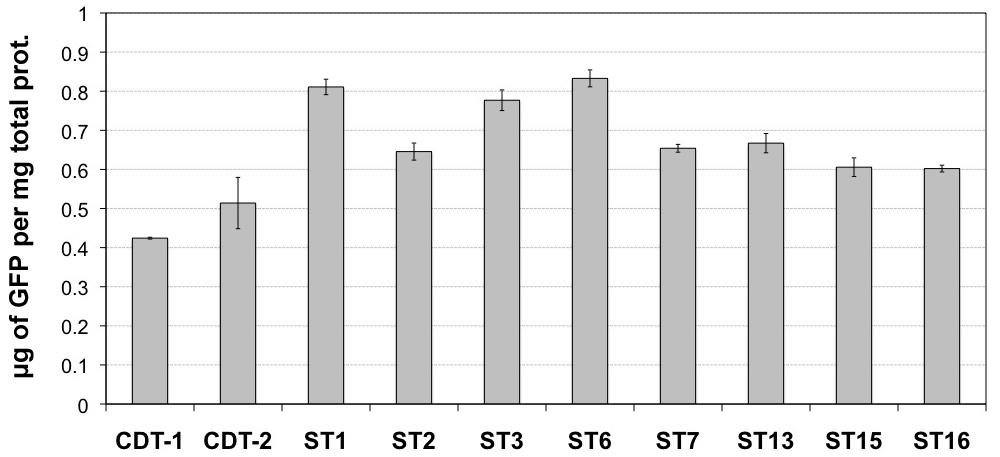

Supplement: S5 Fig — D452-2 cells carrying plasmids containing the transporter-GFP fusions indicated were grown in oMM-Ura+2%G media. Cells were lysed and the clarified supernatants were used to determine transporter concentrations via GFP fluorescence. Total protein concentrations in the lysate were measured using BCA protein assay. GFP protein concentrations represent the mean from 3 biological replicates. (TIFF) [file pone.0184730.s005.tiff]
