# Supplementary material for: Screening of transporters to improve xylodextrin utilization in the yeast Saccharomyces cerevisiae
Source: PLoS One. 2017 Sep 8;12(9):e0184730. doi: 10.1371/journal.pone.0184730 (PMC5591001; doi:10.1371/journal.pone.0184730)
Supplement: S2 Table — (DOCX) [file pone.0184730.s007.docx]

**S2 Table. Advantages and Disadvantages of Select Transporters**

| **Name** | **Advantages** | **Disadvantages** |
| --- | --- | --- |
| ST2 | Fast aerobic growth on XD | Not XD specific; xylobiose transport inhibited by presence of cellobiose |
| ST15 | XD specific transporter; Xylobiose transport is not inhibited by cellobiose | Slow aerobic growth on XD |
| ST16 | XD specific transporter; Fast aerobic growth on XD; Xylobiose transport is not inhibited by cellobiose | Not able to transport xylotriose |
